# Hyperprogressive disease in lung metastases without target lesion progression after durvalumab consolidation therapy: A case report

**DOI:** 10.1111/1759-7714.15104

**Published:** 2023-09-12

**Authors:** Kosuke Masuda, Yoshiaki Nagai, Hikari Amari, Hiroki Tahara, Yuki Maeda, Jun Shiihara, Hiromitsu Ohta, Masahiro Hiruta, Yasuhiro Yamaguchi

**Affiliations:** ^1^ Division of Respiratory Medicine, Clinical Department of Internal Medicine Jichi Medical University Saitama Medical Center Saitama Japan; ^2^ Department of Pathology Jichi Medical University Saitama Medical Center Saitama Japan

**Keywords:** hyperprogressive disease, immune checkpoint inhibitor, lung cancer

## Abstract

Hyperprogressive disease (HPD) is a novel progressive pattern that occurs after immune checkpoint inhibitor (ICI) administration. Here, a 74‐year‐old woman who had undergone right lower lobectomy for lung cancer received curative chemoradiotherapy followed by consolidation therapy with durvalumab for metastatic recurrence confined to the mediastinal lymph nodes. Three weeks later, multiple randomly distributed nodular shadows appeared on chest CT, and thoracoscopic lung biopsy led to the diagnosis of multiple pulmonary metastases. HPD may be suspected when multiple metastases appear in new organs early after the administration of ICIs. This phenomenon may occur not only with ICI monotherapy but also with the administration of ICIs after chemoradiotherapy. Therefore, patients who have received radiation therapy should also be given similar attention early after the administration of ICIs.

## Graphical abstract

We present a unique case of hyperprogressive disease (HPD) of lung cancer. The patient rapidly developed multiple lung metastases without target‐lesion progression 3 weeks after the introduction of durvalumab following curative chemoradiotherapy for postoperative mediastinal lymph node recurrence of lung cancer. HPD may be suspected when multiple metastases appear in the new organs early after the administration of immune‐checkpoint inhibitors.

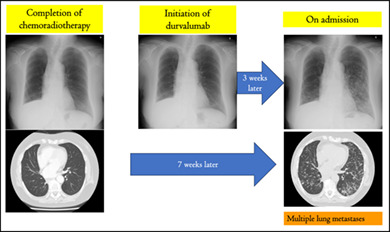

## INTRODUCTION

Lung cancer treatment has been revolutionized by the use of immune checkpoint inhibitors (ICIs), which are now considered a standard therapy, offering a dramatic improvement in long‐term survival.^1^, ^2^, ^3^ However, recent studies have identified a paradoxical acceleration of tumor growth after the administration of ICIs, known as hyperprogressive disease (HPD). There have been few reports of HPD with new multiple metastases, and there is limited information on the occurrence of HPD after chemoradiotherapy.

## CASE REPORT

**FIGURE 1:**
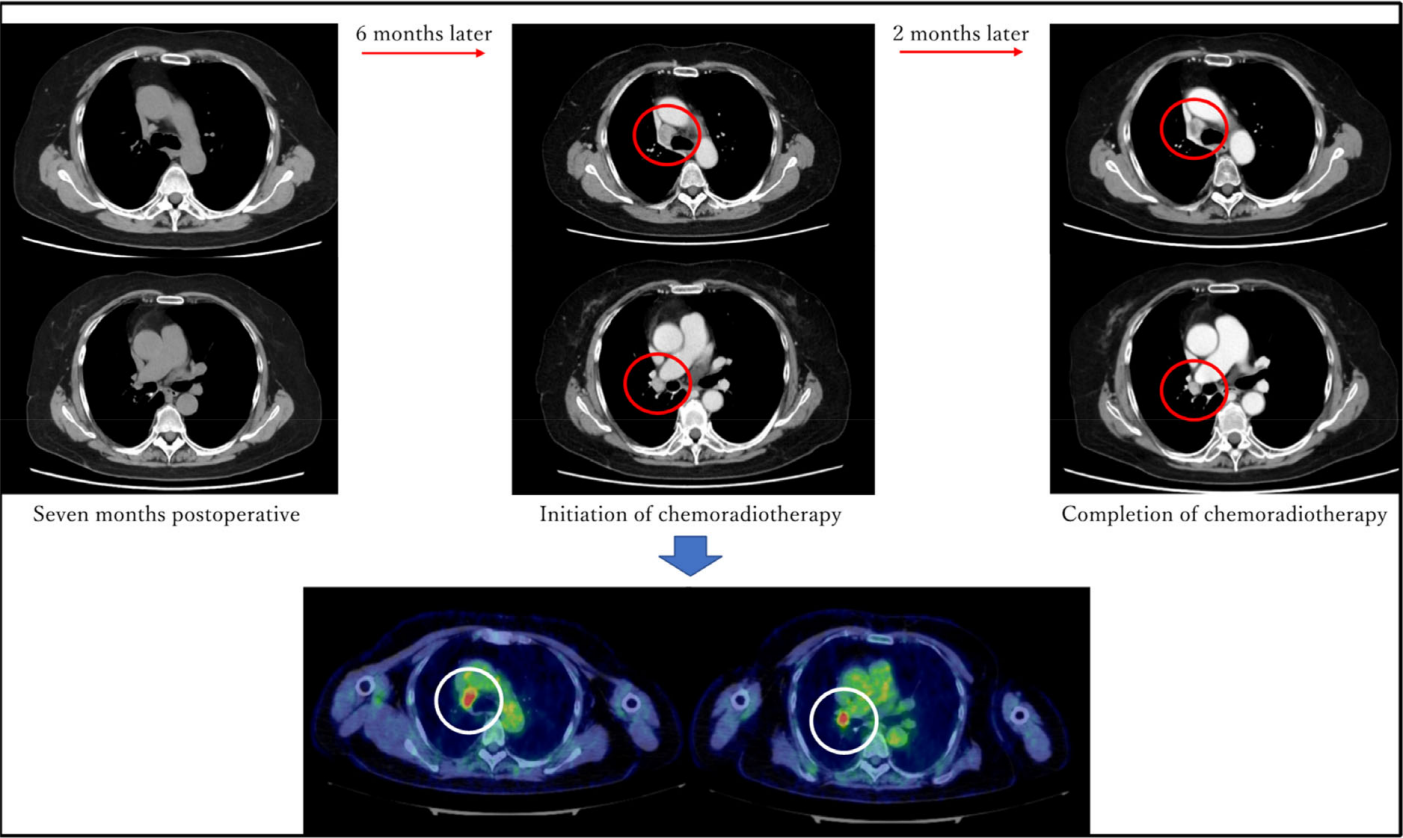
At 7 months postoperatively, no evidence of recurrent disease was seen on chest plain computed tomography (CT) (upper‐left). Chest contrast‐enhanced CT conducted ~6 months later showed the enlargement of two mediastinal lymph nodes (upper center). At approximately the same time, fluorodeoxyglucose positron emission tomography‐computed tomography (FDG/PET‐CT) showed the accumulation of FDG in the same region with an SUVmax of 4.9–5.58 (lower center). Chemoradiotherapy was administered to the same region, and at the time of completion, the disease was stable according to the Response Evaluation Criteria in Solid Tumors criteria (upper‐right).

**FIGURE 2:**
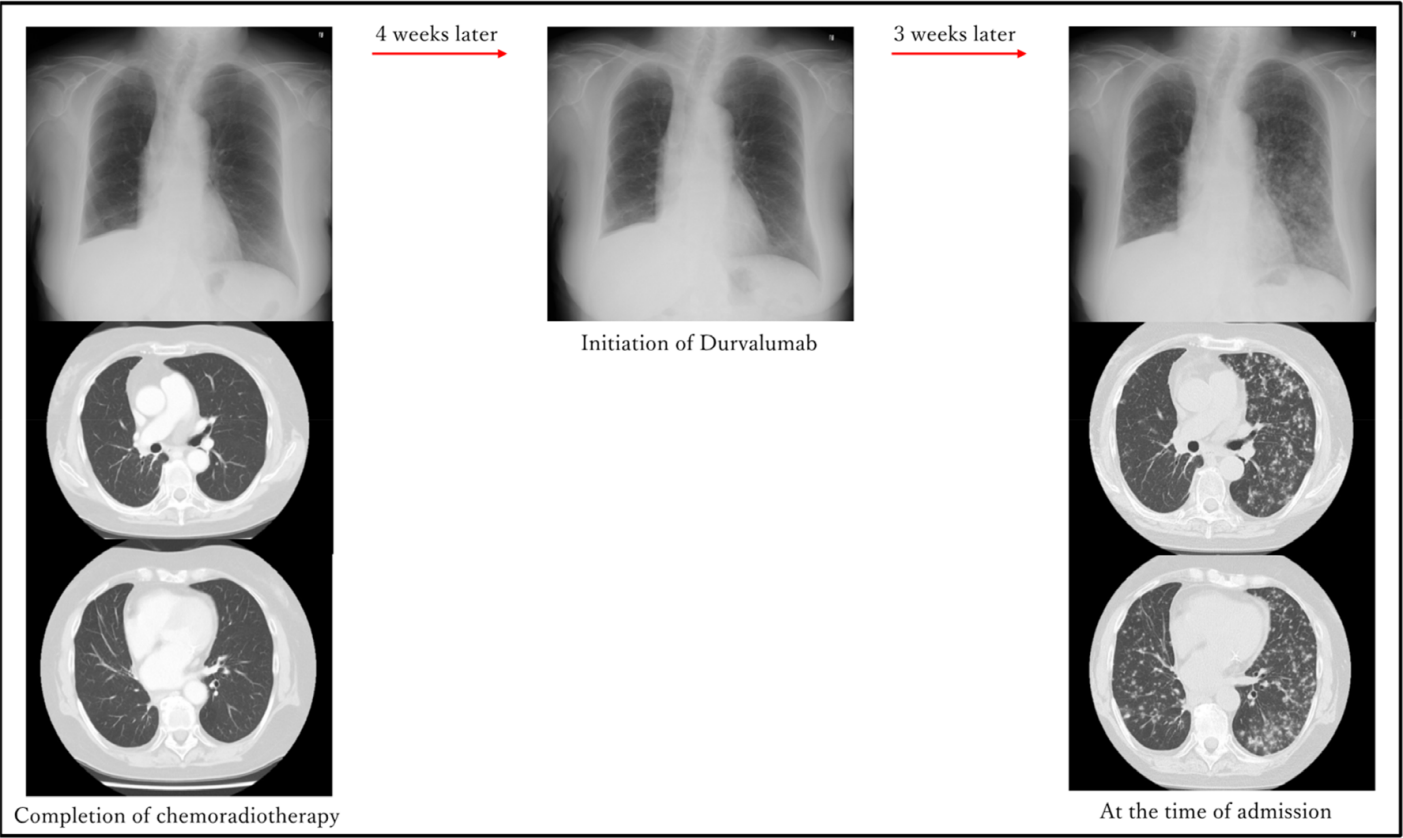
Chest X‐ray and computed tomography (CT) images obtained at the completion of chemoradiotherapy are shown on the left. A chest X‐ray image obtained at the introduction of durvalumab did not show any new abnormal shadows (middle); however, chest X‐ray and CT images obtained 3 weeks later showed multiple randomly distributed granular shadows that were suspected to be multiple lung metastases (right).

**FIGURE 3:**
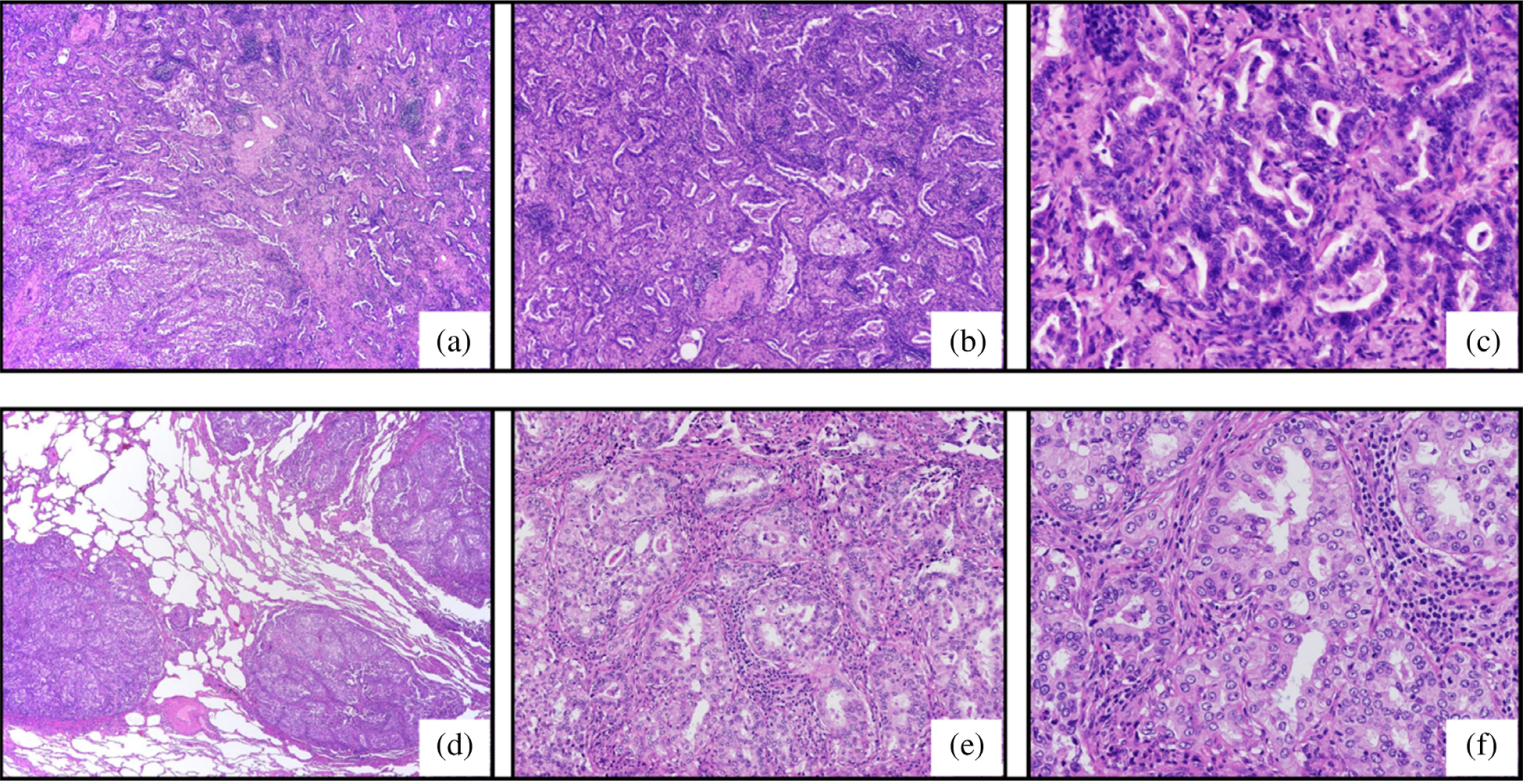
Histopathological findings. The adenocarcinoma has multiple nodular structures, with mild clustering of lymphocytes in the vicinity, in the tissue sample obtained during the previous right lower lobectomy (a–c) and surgical biopsy of the multiple lung nodules after durvalumab consolidation therapy (d–f). Hematoxylin and eosin staining; original magnification ×20 (a, d), ×100 (b, e), and ×200 (c, f).

A 74‐year‐old woman with no prior history of smoking underwent thoracoscopic right lower lobectomy after being diagnosed with primary lung cancer. A postoperative pathological evaluation revealed p‐Stage IB disease. The genetic profile showed Erb‐B2 receptor tyrosine kinase 2 positivity, and the programmed cell death ligand 1 (PD‐L1) expression rate, as determined by the tumor proportion score using PD‐L1 immunohistochemistry 22C3, was 1%–24%. Thirteen months later, recurrence was detected in the mediastinal lymph nodes, but fluorodeoxyglucose positron emission tomography/computed tomography (PET‐CT) and magnetic resonance imaging showed no signs of metastasis in other organs. As a result, curative chemoradiotherapy was performed, followed by consolidation therapy with durvalumab. Three weeks after the initial administration of durvalumab, multiple randomly distributed nodular and nodular infiltrates were detected on chest CT, and the patient was admitted for further investigation and treatment. With the exception of mild tachycardia, her vital signs were within the normal limits. The laboratory data at the time of admission are summarized in Table 1. The carcinoembryonic antigen level, which was 9.9 mg/dL 2 months previously, was markedly elevated at 27.7 mg/dL on admission. The imaging findings during the course are shown in Figures 1 and 2. During bronchoscopy, transbronchial lung biopsy was performed at nine random sites, but all showed only mild inflammatory changes with lymphocytic infiltration. Subsequently, video‐assisted thoracic surgery (VATS) was performed to obtain a definitive tissue diagnosis. A pathological examination revealed the presence of multiple adenocarcinoma nodules, with a maximum diameter of ~6 mm, and mild lymphocytic clustering in the surrounding tissue. The adenocarcinoma nodules appeared to be similar to the tissue sample obtained during the previous right lower lobectomy, confirming the diagnosis of multiple pulmonary metastases of lung adenocarcinoma (Figure 3).

**TABLE 1:** Laboratory data on admission.

Blood					
Parameters	Values	Reference range	Parameters	Values	Reference range
White blood cell count, /*μ* L	7060	6600–8100	Chloride, mEq/L	105	100–110
Neutrophil, %	65.5	40–74	C‐reactive protein, mg/dL	0.68	<0.14
Eosinophil, %	9.9	0–7	Brain natriuretic peptide, pg/mL	27.3	<18.4
Red blood cell count, × 10^4^/*μ* L	312	376–500	Erythrocyte sedimentation rate, mm	99	<15
Hemoglobin, g/dL	10	11.3–15.2	Angiotensin converting enzyme, IU/L	11.2	8.3–21.4
Platelet count, × 10^3^/*μ* L	35.6	13.0–36.9	Rheumatoid factor, IU/mL	<10	<15
PT, %	>100	70–140	Antinuclear antibody	<40×	
PT‐INR	0.97	0.9–1.2	Anti‐aminoacyl‐tRNA synthetase antibody	Negative	
Total protein, g/dL	6.7	6.6–8.1	MPO‐ANCA, U/mL	<1.0	<3.5
Albmin, g/dL	3.4	4.1–5.1	PR3‐ANCA, U/mL	<1.0	<3.5
Total bilirubin, mg/dL	0.33	0.4–1.5	IgG, mg/dL	1321	870–1700
Aspartate aminotransferase, U/L	30	13–30	IgA, mg/dL	235	110–410
Alanine aminotransferase, U/L	23	7–23	IgM, mg/dL	38	46–260
Lactate dehydrogenase, U/L	189	124–222	IgG4, mg/dL	70.6	11–121
Creatine kinase, U/L	41	41–153	Carcinoembryonic antigen, ng/mL	27.7	<5.0
Urea nitrogen, mg/dL	10	8–20	Soluble interleukin‐2 receptor	843	157–474
Creatinine, mg/dL	0.63	0.46–0.79	Beta‐D‐glucan	5.9	<20
Uric acid, mg/dL	5.5	2.6–5.5	Tuberculosis specific interferon‐*γ*	Negative	
Sodium, mEq/L	139	138–145	Cryptococcus neoformans antigen	Negative	
Potassium, mEq/L	4.1	3.6–4.8	Cytomegalovirus pp65 antigen (C10, C11)	Negative	

Abbreviations: Ig, immunoglobulin; KL‐6, Krebs von den Lungen‐6; MPO, myeloperoxidase, PR3, proteinase3; PT, prothrombin time; PT‐INR, prothrombin time‐international normalized ratio.

## DISCUSSION

We identified two clinically important issues. First, HPD may appear as multiple metastases to new organs, even if there is no increase in the size of the target lesions after treatment with ICIs. Many reported cases of HPD have been defined based on the tumor volume or the speed of increase in target lesions using tumor growth kinetics, tumor growth rate, or Response Evaluation Criteria in Solid Tumors. Petrova et al. defined HPD based on the presence of three of the following criteria: progression‐free survival of <3 months, a ≥50% total increase in the longest diameter of the target lesion, at least two new lesions appearing in organs where there were already lesions, spread of the disease to a new organ, and clinical deterioration with an Eastern Cooperative Oncology Group (ECOG) performance status (PS) of ≥2 within 3 months after the initiation of treatment.^4^ Although this case showed new metastasis to distant organs 3 weeks after the initiation of treatment, at the time of writing this report, the target lesions had not changed, and the ECOG PS was 1. Therefore, the present case does not strictly meet the abovementioned definition. However, the pathophysiology of HPD is still unclear, and there is no unified definition. Tariq et al. reported a case in which multiple brain metastases and left adrenal gland metastasis appeared as HPD, despite a reduction in the target lesion size, 4 weeks after the introduction of durvalumab following chemoradiotherapy.^5^ Further elucidation of the pathophysiology and a more refined definition of HPD are needed to determine whether these cases are truly HPD.

Second, even when ICIs are administered as consolidation therapy after chemoradiotherapy, HPD may occur. In lung cancer chemotherapy, ICIs are often used for the treatment of lung cancer with metastasis, but they have recently been approved for consolidation therapy after curative chemoradiotherapy for locally advanced unresectable non‐small cell lung cancer.^3^ Regarding the relationship between radiotherapy and HPD, Ocak et al. reported that radiotherapy may suppress the occurrence of HPD.^6^ In addition, reports have shown that radiotherapy can activate the immune system by releasing tumor antigens,^7^ and after antigen release, dendritic cells take up the antigens and present them to effector T cells, serving as a bridge for systemic immune responses.^8^ Zhao et al. concluded that the induction of sufficient antigen release by radiotherapy helps to suppress the occurrence of HPD.^9^ On the other hand, radiotherapy may stimulate the endogenous expression of PD‐L1 through the induction of IFN‐I secretion.^10^ This mechanism would suppress HPD more in the target lesion than in the nontarget lesion, causing discrepancy between the target lesion and new metastasis. To our knowledge, there is only one reported case of HPD in which new distant metastases appeared despite the tumor at the original site not increasing in size.^5^ Importantly, in that case, the peculiar HPD occurred just after the patient received ICI subsequent to chemoradiotherapy. Therefore, when administering ICIs to patients who have received radiation therapy, attention is required to detect the sudden development of multiple distant metastases early after ICI administration.

## AUTHOR CONTRIBUTION

Conceptualization, Kousuke Masuda, Yoshiaki Nagai, Hiromitsu Ohta; data curation, Kousuke Masuda, Yoshiaki Nagai, Hikari Amari, Hiroki Tahara, Yuki Maeda, Jun Shiihara, Hiromitsu Ohta, Masahiro Hiruta, Yasuhiro Yamaguchi; writing–original draft preparation, Kousuke Masuda; writing–review and editing, Yoshiaki Nagai, Jun Shiihara, Yasuhiro Yamaguchi; visualization, Kousuke Masuda, Yoshiaki Nagai, Masahiro Hiruta, Yasuhiro Yamaguchi; supervision, Yoshiaki Nagai, Jun Shiihara, Hiromitsu Ohta, Yasuhiro Yamaguchi. All authors discussed the results and commented on the manuscript.

## CONFLICT OF INTEREST STATEMENT

The authors have no Conflict of Interest.
